# Supplementary material for: The Construction and Exploration of a Comprehensive MicroRNA Centered Regulatory Network in Foxtail Millet (Setaria italica L.)
Source: Front Plant Sci. 2022 May 6;13:848474. doi: 10.3389/fpls.2022.848474 (PMC9121102; doi:10.3389/fpls.2022.848474)
Supplement: Supplementary file 3 [file Data_Sheet_3.zip › Sit-miRNovel5_Seita.3G029800.1_918_TPlot.pdf]

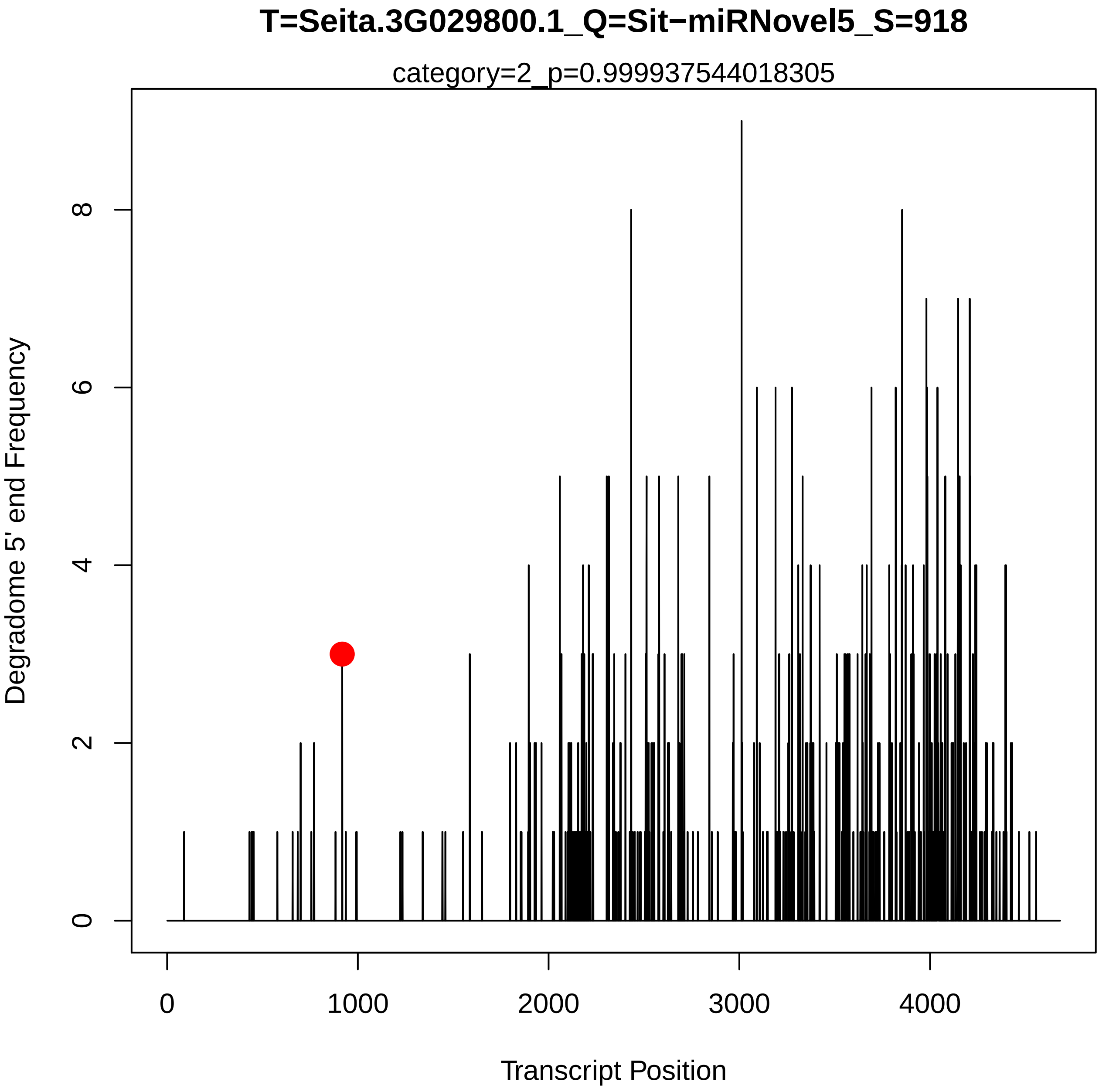
T=Seita.3G029800.1_Q=Sit−miRNovel5_S=918
category=2_p=0.999937544018305
Degradome 5' end Frequency
0
2
4
6
8
Transcript Position
0
1000
2000
3000
4000
